# Application and toxicity studies of arabinoxylan and β-d-glucan stearic acid ester composite coatings in extending postharvest storage of peach

**DOI:** 10.1038/s41598-021-03163-5

**Published:** 2021-12-13

**Authors:** Usman Ali, Prabhjot Kaur, Swati Kanwar, Vibhu Kumar, Rohit Maurya, Mahendra Bishnoi, Santanu Basu, Koushik Mazumder

**Affiliations:** 1 National Agri-Food Biotechnology Institute, Sector-81 (Knowledge City), S.A.S. Nagar, Mohali, Punjab 140306 India; 2 Department of Molecular Sciences, Swedish University of Agricultural Sciences, P.O. Box 7015, 750 07 Uppsala, Sweden

**Keywords:** Chemistry, Materials science

## Abstract

Peaches are good source of nutrients and known for their taste and aroma. The highly perishable nature of the peaches tends to decay rapidly during transportation and storage is a serious constraint for efficient transportation and storage. Therefore, the effect of arabinoxylan (AX) and β-D-glucan stearic acid ester (SABG) composite coating material was examined for the postharvest storage quality of peach under storage at 22 ± 2 °C with 85% relative humidity (RH). Both, AX-SABG and shellac (1–2%) coatings significantly reduced the change in the quality attributes like weight loss (1.2–1.4 fold), respiration rate (1.1–1.2 fold), ripening index (1.3–1.5 fold) and firmness (1.3–1.5 fold) during 6 days storage as compared to the uncoated peaches. In addition, AX-SABG (1–2%) coating was more effective in retaining aroma volatiles and reducing disease incidence compared to shellac. Further, acute and chronic toxicological studies have shown no tissue related toxicity and mortality in mice. Our results suggest that AX-SABG as an edible coating has the potential to preserve the fruit quality during 6 days storage at 22 ± 2 °C and extend the postharvest shelf life of peach during storage.

## Introduction

Peach (*Prunus persica* L.) is a climacteric fruit grown widely in northern region of India. The quick ripening and quick softening after harvesting of the fruit is responsible for its shorter shelf-life and subsequent fruit spoilage. The fast ripening is a serious constraint in efficient postharvest handling, storage and transportation during marketing chain^1^. Different studies showed the high (18.31%) postharvest lossesin peach due to high perishability^2^. Different approaches such as cold storage, controlled and modified atmospheric storage have been used to increase the postharvest shelf life of perishable fresh fruits. But, these techniques are not very cost effective in developing and under developed countries^3,4^. Edible coating is a cost effective and simple technology, which can be used to reduce the postharvest losses in fruit**s** during storage. Edible coating performs as a barrier against the gases, moisture and solute movement by developing a semi-permeable membrane on the surface of the fruits. Thus the coating material suppress the respiration rate, retard dehydration, retain the volatile compounds, improve firmness of the fruits and slow down the oxidation process^5,6^. Different types of coating materials have been used to enhance the postharvest shelf life of peach^7,8^. Due to hydrophilic nature, polysaccharide based coatings have poor moisture barrier properties. On the other hands the wax based coating are being questionable due to effect of wax on human health^9^. Several studies have demonstrated that incorporation of lipid components (fatty acids) to polysaccharide (hydroxypropyl-methylcellulose (HPMC), sodium caseinate and starch based films) and protein based films were beneficial to modulate the moisture barrier properties^9–12^.

The literature reveals that most of the coating materials used for peach were based on the polysaccharides such as carboxymethyl cellulose, starch and alginate. These are not an effective coating materials due to hydrophilic character and the hydrophobic coating cause the reduced permeability and fermentation issue^8,12,13^. The present study is focused on the development of composite (hydrophilic and hydrophobic) edible coating material based on wheat straw arabinoxylan and β-d-glucan stearic acid ester. There is no literature has been yet reported on the application of arabinoxylan and β-d-glucan stearic acid ester coating to increase the postharvest shelf life of highly perishable fruit crop such as peach.

Several studies have reported that edible coating (1–2%) based on carboxymethyl cellulose, chitosan, alginate and rhubarb extract retarded the postharvest ripening of peach during storage^1,14,15^. In another study chitosan chlorogenic acid conjugate acted as a potential preservative agent or coating material during postharvest storage of peach^16^. Our previous study also demonstrated that incorporation of β-d-glucan stearic acid ester to wheat straw AX film significantly reduced the water vapor permeability to nearly 67% suggesting improvement in the moisture barrier properties^17^. Application of AX-SABG (1–2%) with high emulsion stability remarkably improved the postharvest quality of apple^18,19^. Therefore, AX-SABG (1–2%) was used to determine the efficacy of coating material in comparison to commercially available shellac (1–2%) coating on highly perishable climacteric fruits (Peach) under super-market storage conditions (22 ± 2 °C with 85% RH)^7,20^. The present study aimed to explore the effect of wheat straw arabinoxylan and β-d-glucan fatty acid ester coatings on postharvest quality and shelf life of the peach during storage.

## Materials and methods

### Raw material

#### Plant materials

Arabinoxylan and β-d-glucan were extracted from the wheat straw and oat bran. The present study involving the plant materials complies with the relevant institutional, national and international guidelines. Further, the study does not involve the use of specific plant materials from experimental area. In our study commercially available wheat straw (HD 2967) was collected from local agricultural filed at Mohali, Punjab. The wheat variety was released by Panjab Agricultural University (PAU), Ludhiana. The oat seed (Haryana Javi-8) was procured from Chaudhary Charan Singh Haryana Agricultural University (CCSHAU), Hisar, Haryana.

#### Fruit sample and materials

Peaches (*Sharbati*) with uniform shape, size and maturity were obtained from a local orchard (Mohali, Punjab, India). Peaches were packed in cardboard corrugated boxes and transported to laboratory within 4 h after harvesting by a commercial vehicle. After that, peaches were washed with clean water and sanitized with sodium hypochlorite (100 ppm) for 2 min and subsequently rinsed with clean water. Commercial shellac, dialysis tube, and all other chemicals were procured from the Sigma-Aldrich (USA).

### Preparation of coating formulation

Arabinoxylan (AX) and β-d-glucan stearic acid ester (SABG) were produced from wheat straw and oat β-D-glucan as previously described^18,19^.

For the preparation of coating formulation, AX and SABG were homogenized at room temperature in aqueous-ethanol (80:20, v/v) solution containing glycerol (20% v/w) as a plasticizer by using ultra homogenizer (IKA T25, Germany)^21^. The purpose of adding alcohol is to reduce the drying time. Shellac (1% and 2%) coating solution was prepared by dissolving in ammonium hydroxide solution (0.5%) at 95 °C as described previously^22^.

### Experimental design

The experiment was performed under controlled storage condition. The uncoated and coated (AX-SABG and shellac) peaches (25 fruits in each group) were stored under recommended super-market storage conditions (22 ± 2 °C with 85% relative humidity) (Percival Scientific, USA)^7,20^. The postharvest storage quality parameters of uncoated and coated (AX-SABG and shellac) peaches were determined during 6 days storage.

### Application of AX-SABG and commercial (shellac) coatings

The AX-SABG (1–2%) and commercially available shellac (1–2%) coatings were used for the surface coating of peaches by using latex gloved hands method of coating^23^. Briefly, 1 ml of the coating material per fruit was spread uniformly on the surface of the fruit and finally dried for 15 min by blowing air at room temperature.

### Weight loss

Weight loss was determined by weighing the peach (25 peaches in each group) after storage. The weight loss percent was calculated with respect to initial weight using balance (PS 3500/C/2, Radwag, EU).

### Respiration rate

Respiration rate of peaches were determined by placing the fruits (with known quantity and weights) in an airtight container as previously described by Maftoonazad et al.^8^. The concentration of CO_2_ was monitored by placing a CO_2_ sensitive sensor (CheckMate3, PBI Dansensor, Denmark) inside of the container. Respiration rate was calculated by using the regression slope of concentration of CO_2_ against time. The results were expressed as ml CO_2_/kg h.

### Color

Color parameters (L*, a* and b*) of fruits were obtained individually for each fruit using Hunter Lab Colorimeter (Color Flex EZ, Hunter Lab, USA) from different side of fruits^7^. The color parameters (a* and b*) was further used to determine the hue angle^24^ calculated as:

$${\text{Hue angle}}\, = \,{\text{arctangent }}({\text{b}}^{*}/{\text{a}}^{*}).$$

### Firmness

The firmness of peach fruit was measured by using texture analyzer (TA-HD plus, Stable Micro System, UK)^25^. Fruits were penetrated at opposite point on peach equator using a 10 mm diameter probe. Force obtained from force–deformation curve was expressed as the force–deformation in Newton (N).

### Total soluble solids, titratable acidity and ripening index

Total soluble solids (TSS) in peach juice samples were determined by using a digital refractometer Atago PR-101 (Atago Co. Ltd., Tokyo, Japan) at 20 ºC, and results were expressed as % (^°^Brix). Total acidity (TA) was determined by titrating 1 ml peach juice with 0.1 N NaOH solution, upto pH 8.1. The results were presented as g malic acid equivalent per 100 g^−1^ fresh weight^7^.

The ripening index (RI) was obtained as a ratio of TSS and TA^7^.

### Enzyme activity

The enzymatic (polyphenol oxidase) activity of the peaches were determined as described previously^26,27^. The enzyme extraction solution was prepared in 0.2 M sodium phosphate buffer at pH 6.5 containing 4% (w/v) poly(vinylpyrrolidone) and 1% (v/v) Triton X-100. The homogenized sample (10 g) of peaches was mixed with 20 ml of extraction solution. The extraction mixture was homogenized using ultra-homogenizer at 4 °C for 3 min and centrifuged at 4000 rpm for 10 min. The supernatant of the reaction mixture was used to determine the enzyme activity.

### Polyphenol oxidase activity (PPO)

For the measurement of the PPO activity, enzyme extract (75 µl) was mixed with 3 ml of 0.05 M sodium phosphate buffer (pH 6.5) solution containing 0.07 M catechol. Distilled water used for the preparation of blank instead of the enzyme extract. Absorbance of assay mixture was measured in a kinetic mode at 420 nm and 25 °C for 10 min using a spectrophotometer (Spectra Max M5^e^, China). The activity of the enzyme was expressed as the alteration of absorbance/min/g fresh weight of the sample^27^.

### Determination of volatile compounds

Gas chromatography coupled with flame ionization and mass spectrometry detectors were used for the estimation of volatile compounds in peach^23,28,29^ (Agilent Technologies 7890, USA). Briefly, 14 g of peach pulp sample was homogenized with saturated NaCl solution (6 ml) using homogenizer (IKA T25, Germany). Prior to the analysis, 4 ml of the homogenate in 10 ml sample vial sealed with teflon coated septum were stored at −80 °C. For analysis, sample vials were thawed under tap water, after thawing the homogenate heated in water bath at 80 °C. The solid phase micro extraction (SPME) SPME fiber was exposed for 30 min into the headspace of sample vial.

Thereafter, the SPME fiber was desorbed via split less mode for compound identification on to DB-5 (60 m × 0.25 mm, 1.00 μm film thickness, Agilent) column. The analysis was performed by using oven temperature gradient: 40 °C for 2 min, 40–100 °C at 5 °C/min, 100–230 °C at 4 °C/min, 230–250 °C at 5 °C/min. The mass spectra library of NIST (National Institute of Standards and Technology, USA) was used for the identification of the volatile compounds and the retention time was compared with the standards. Regression equations was used for the calculation of the concentrations measured by injecting each standard in five different concentrations to obtain a calibration curve^23^.

### Determination of disease of incidence

The percentage of decay incidence was determined with respect to the total number of fruits per treatment in uncoated and coated fruits stored at 85% RH at 22 °C (± 2)^30^.

### Animal experiments

The animal experiments were carried out in compliance with the ARRIVE guidelines & ethical guidelines of Institutional Animal Ethical Committee (IAEC) and Committee for the purpose of control and supervision of experiments in animals (CPCSEA). The ethical approval (Approval no. PU/45/99/CPCSEA/IAEC/2018/219 and IAEC/2019/15) was obtained from Institutional Animal Ethical Committee, Panjab University Chandigarh and NABI Mohali. All experimental protocols/procedures were performed according to the committee and Indian National Science Academy guidelines for use and care of experimental animals. Swiss albino mice (25–35 g, 5–6 weeks old, male) were used from the breeding facility of NIPER, Mohali for animal studies. Mice were acclimatized for one week under standard laboratory conditions (Temp: 25 ± 2 °C; humidity: 60%, with 12 h light dark cycle) provided with normal chow diet.

#### Experiment 1: acute oral toxicity study

14-day acute toxicity study was performed according to OECD guideline 423^31^. The mice were divided into 5 groups (n = 6) and fed with fixed single oral dose of AX-SABG. Group 1 served as control, Group 2, 3, 4 and 5 were administered with AX-SABG at 50, 300, 2000 and 5000 mg/kg bodyweight (bw) respectively. After oral dosage, feed was withheld for 1–2 h. The mice were observed for mortality or any signs of toxicity for the period of 14 days. The body weight changes and feed/water intake per cage during 24 h was noted down. The mice were sacrificed by cervical dislocation on the last day of the experiment and tissues (colon, liver and kidney) were excised for further histological studies.

#### Experiment 2: sub-acute oral toxicity study

28-day sub-acute oral toxicity study was performed in accordance with OECD guideline 407^32^. The daily dosage was calculated according to the equivalent amount of AX-SABG required to coat 3 fruits and administered once daily for 28 days by oral gavage at volume 10 ml/kg bw. Animals were randomly assigned to five experimental groups (n = 6): Group 1 served as control, Group 2 was fed with AX and Group 3 with SABG. While, Group 4 and Group 5 were fed with 1% and 2% AX-SABG coating formulation (60:40, w/w) containing glycerol (20% of the total dry material) respectively. During the experimental period, mice were observed for clinical signs including mortality, or any other signs of toxicity twice daily till sacrifice^33^.

### Histological studies

Histological studies were conducted to observe any morphological changes in the liver, colon and kidney of treated groups. Tissue samples of control and treated mice from each group were removed and fixed in 10% formalin at room temperature. The samples were serially dehydrated in ethanol gradient and then replaced by xylene and molten paraffin. After paraffin embedding, the tissues were cut into ultrathin sections (0.5 μm) using microtome (Leica RM2255, Leica Biosystems, Germany) and mounted on glass slides^34^. Haematoxylin–Eosin (HE) staining was performed according to^35^ to visualize the morphology of the tissues. The permanent slide of the tissues was examined under bright field microscope (Leica Biosystems, Germany). Quantitative analysis of the toxicological parameters of stained slide was done using ImageJ software^36^. Six measurements were made randomly from each sample in triplicates for each parameter and the mean value was used for statistical analysis.

### Statistical analysis

All the experiments were conducted in triplicate. The experimental data were subjected to analysis of variance (ANOVA) by Prism Graph Pad 7 software. Tukey’s multiple comparisons tests was used for the determination of differences between means at a significant level (*P* < 0.05). Furthermore, MeV (Multi Experiment Viewer) version 4.9.0. and ClustVis a web tool (https://biit.cs.ut.ee/clustvis/) were used for Heatmap and Principal component analysis (PCA) respectively.

## Results and discussion

### Weight loss and respiration rate

**Figure 1:**
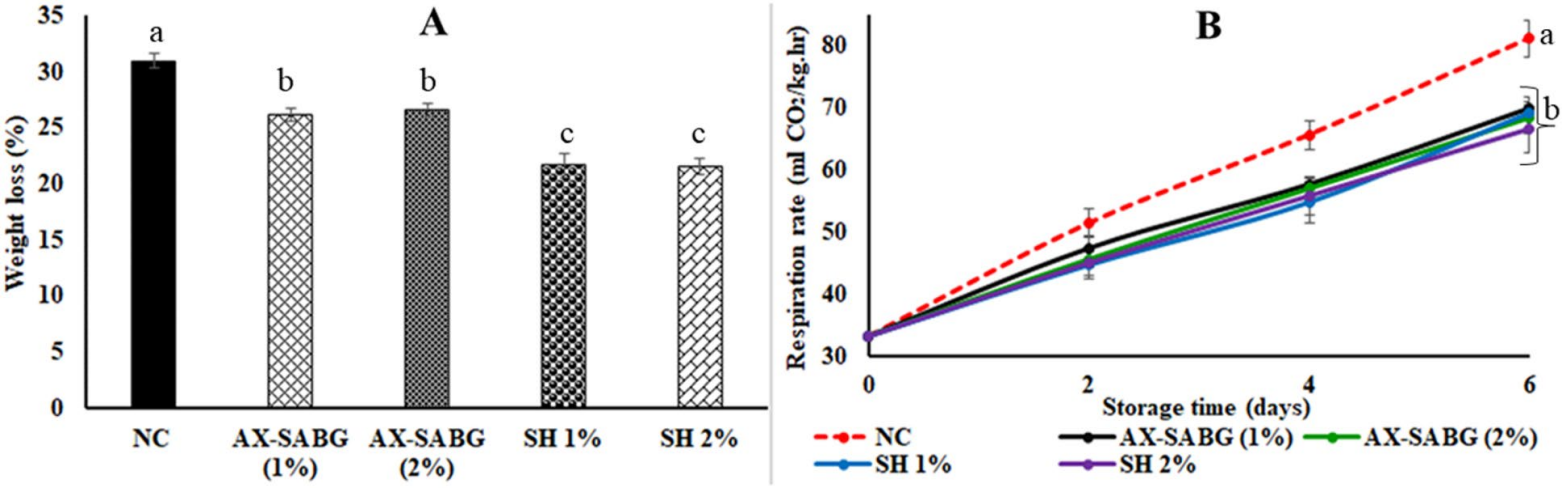
**(A)** Change in weight loss (%) and **(B)** respiration rate (ml/CO_2_/kg h) of peaches during storage (*NC* uncoated, *AX-SABG* arabinoxylan-β-d-glucan stearic acid ester, *SH* shellac). Data were analysed using ANOVA followed by Tukey’s multiple comparisons tests. The letters compare changes between the treatments. Different letters indicate significant differences at *p* < 0.05.

Application of AX-SABG and shellac as an edible coating had a significant effect on reducing the moisture loss of peach during storage. The moisture loss was significantly (*P* < 0.05) higher in uncoated peaches (30.9%) as compared to the coated (AX-SABG and shellac) peaches after 6 days storage (Fig. 1A). The peaches coated with AX-SABG and shellac (1–2%) coatings showed nearly 1.2–1.4 fold reduction in moisture loss compared to uncoated peaches under similar storage. The reduced moisture loss in AX-SABG and shellac (1–2%) coated peaches was obviously due to the moisture barrier properties of the coating material. Several studies have reported that, polysaccharide coatings in combination with lipids reduced the moisture loss in coated fruits and vegetables. These coatings act as a physical barrier against the process of transpiration during storage^37^.

Characteristically peach is a climacteric fruit and the climacteric behaviour of the fruit is clearly explained by the respiration rate of the fruit^8^. Increase in the respiration rate of peaches during storage could be linked to the characteristic climacteric process of ripening. It was evident by several studies that as the ripening begins in the climacteric fruits such as peach, apple and banana etc. a dramatic increase in the ethylene production occurs which is concurrent with a rapid increase in the respiration rate^7,25,38^. Further it was suggested that the rise in the respiration rate of the climacteric fruits occurred simultaneously with the increase in the ethylene production or it follows soon afterwards^39–43^. It was also reported that carbon dioxide (CO_2_) had various effect on the ethylene biosynthesis, where CO_2_ was demonstrated as essential co-factor for the key enzymes^44^.The change in the behavior of respiration rate in peaches coated with aloe vera gel has already been demonstrated by Guillén et al.^7^. The respiration rate of both control and treated fruits increased during storage while application of both gels, especially aloe vera, led to a delay in this increase^7^. In the present study, the respiration rate was significantly higher (*P* < 0.05) in the uncoated peaches during 6 days storage. The uncoated peaches showed remarkable rise in the respiration rate of 197.8–244.6% during 4–6 days storage which corresponds to nearly 1.9–2.4 fold rise compared to initial storage. Under similar storage, both AX-SABG and shellac (1–2%) coatings were effective in maintaining the rise in respiration rate by only 1.7–2.1 and 1.6–2.1 folds respectively (Fig. 1B). The study also suggested reduced respiration rate by nearly 1.1–1.2 fold in both AX-SABG and shellac (1–2%) coatings compared to uncoated peaches could be responsible for prevention in moisture loss^7^. The results are also consistent with the previously reported study of surface coatings on peach with sodium alginate and methylcellulose that significantly reduced the respiration rate of coated peaches^8^.

### Color and ripening index

**Figure 2:**
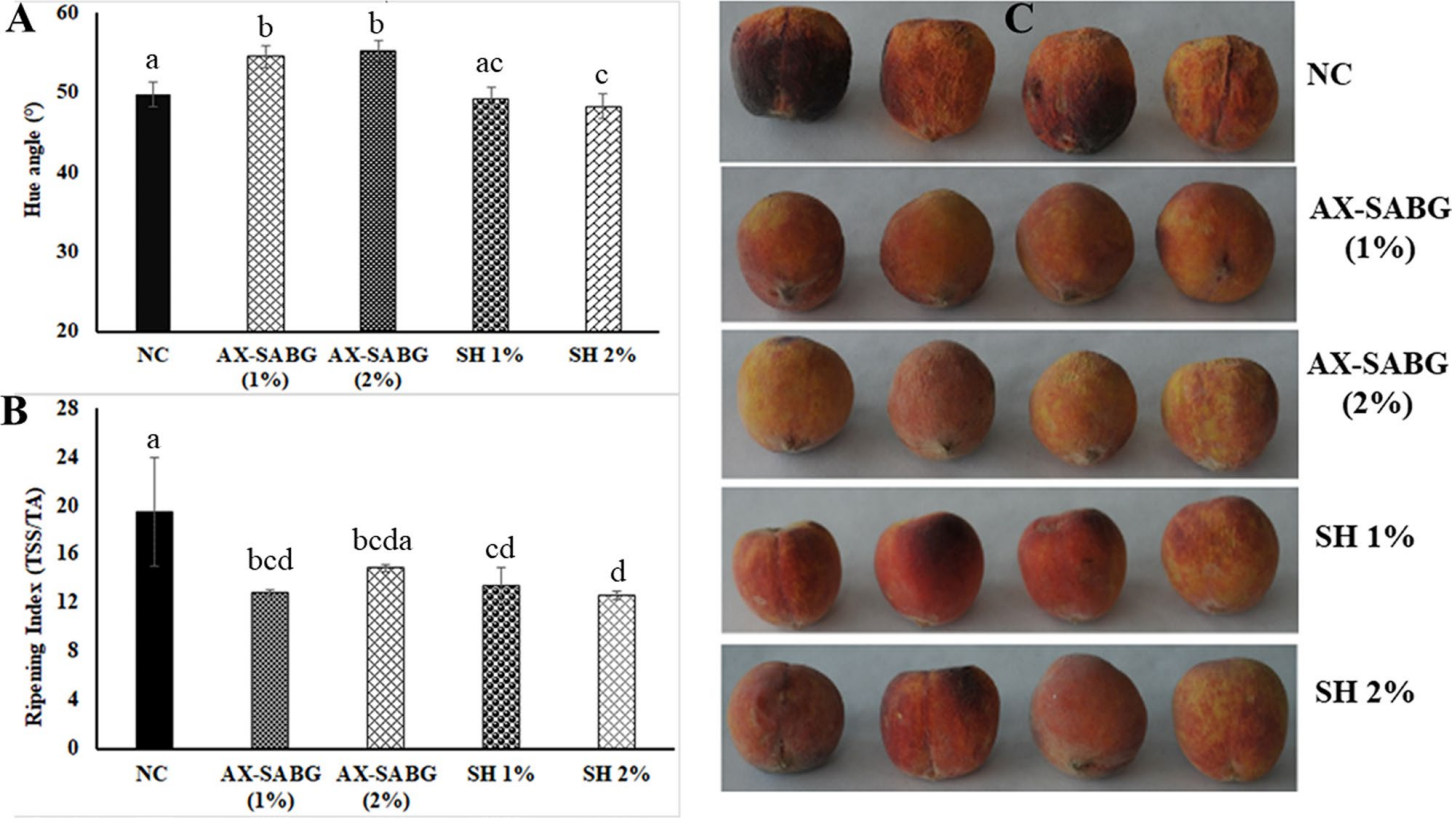
**(A)** Change in color (hue angle), **(B)** ripening index and **(C) **representative images of peaches during storage (*NC* uncoated, *AX-SABG* arabinoxylan-β-d-glucan stearic acid ester, *SH* shellac). Data were analysed using ANOVA followed by Tukey’s multiple comparisons tests. The letters compare changes between the treatments. Different letters indicate significant differences at *p* < 0.05.

Color is the most important appearance characteristic of fruits, being main criteria to determine the maturity of the fruit by the consumer. The changes in the color (Fig. 2C) are associated with alteration in fruit metabolism which is due to chlorophyll degradation and increase in the synthesis of anthocyanin and carotenoids during storage^45,46^. Both uncoated and shellac (1–2%) coated peaches showed notably reduction in the hue values in the range of 49.7 and 48.3–49.1 (Fig. 2A) respectively during 6 days storage. However, peaches coated with AX-SABG (1–2%) exhibited maximum hue values in the range of 54.4–55.1 suggested the high efficacy of AX-SABG coating in maintaining fruit metabolism and chlorophyll degradation^46^. The results were also in agreement with previous studies which suggested lipid based coating were responsible for unacceptable change in color and texture in fruits during storage^47^.

The ratio of total soluble solid and total acidity was expressed as ripening index. The total soluble solid contents of fresh peach were higher during the storage in both uncoated and coated peaches as compared to the fresh peaches. However, the rise in TSS was more prominent in uncoated peaches compared to coated fruits which suggested advancement in the ripening process. After 6 days storage, the uncoated peaches showed a significant increase in the ripening index of 19.5. However, AX-SABG and shellac (1–2%) coated peaches had ripening index in the range of 12.8–14.8 and 12.6–13.4 respectively suggesting nearly 1.3–1.5 and 1.4–1.5 fold reductions compared to uncoated peaches (Fig. 2B). Further, the ability of AX-SABG and shellac in maintaining respiration rate could be linked to the reduction in the ripening index^39^. Previous study also showed that peaches coated with aloe arborescens and aloe vera gel showed the reduced ripening index compared to the uncoated peaches^7^. The effect of coatings on delaying the postharvest ripening of fruit is similar to the previously reported studies on peach, plum, sweet cherry, strawberry, nectarine and papaya^7,13,16,48^.

### Texture and disease incidence

**Figure 3:**
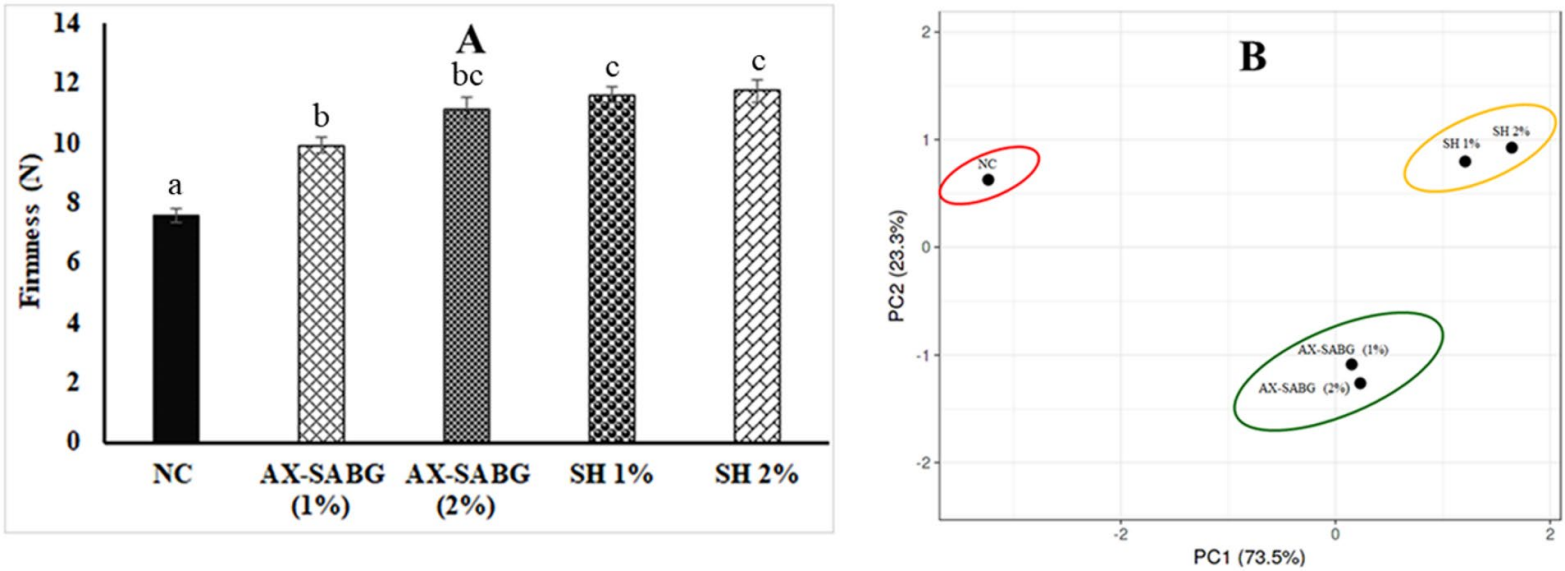
**(A)** Change in firmness and **(B)** PCA of correlation between different properties of peaches during storage (*NC* uncoated, *AX-SABG* arabinoxylan-β-d-glucan stearic acid ester, *SH* shellac). Data were analysed using ANOVA followed by Tukey’s multiple comparisons tests. The letters compare changes between the treatments. Different letters indicate significant differences at *p* < 0.05.

Firmness is an important quality attribute especially during the postharvest storage of peaches. Loss of turgor pressure and moisture from fruits during respiration, transpiration and cell wall hydrolysis were the major factors responsible for the softening of fruits during storage^25^. During 6 days storage, the uncoated peaches had remarkably low fruit firmness of 7.6 N suggested nearly (65.9%) reduction in the firmness compared to initial storage. However, after 6 days storage, AX-SABG coated peaches showed firmness retention in the range of 9.9–11.1 N, whereas shellac (1–2%) coated peaches showed the firmness retention in the range of 11.6–11.7 N (Fig. 3A). Therefore, significantly higher (*P* < 0.05) firmness in the AX-SABG and shellac coated peaches might be due to the reduced respiration and transpiration in the coated peaches. The results were in agreement with previous studies where methyl cellulose and sodium alginate were beneficial in firmness retention of the coated peaches^8^**.** The fruit decay rate was also monitored during storage. The uncoated peaches showed the higher decay rate (~ 8%) compared to the AX-SABG (1–2%) coated peach. The reduced decay rate (~ 4%) in AX-SABG (1–2%) and shellac (1%) peaches might be due to the reduced metabolic activity of coated fruits. Furthermore, significantly higher fruit decay (~ 8%) was observed in shellac (2%) coated peaches which might be due to development of anaerobic respiration^49^.

PCA was used as an explanatory tool for grouping the measured variables and visualizing the positions of all the categories (uncoated and coated peaches) with respect to their properties (Fig. 3B). These variables included the weight loss, respiration rate, hue angle, ripening index and firmness. The two principal components (PC1 and PC2) explained 73.5% and 23.3% of the variances respectively and resulted in a cumulative value of 96.8%. PCA supported the conclusion that AX-SABG (1–2%) coated peaches had higher storability and shelf life compared to uncoated fruits during 6 days storage.

### Polyphenol oxidase activity (PPO)

**Figure 4:**
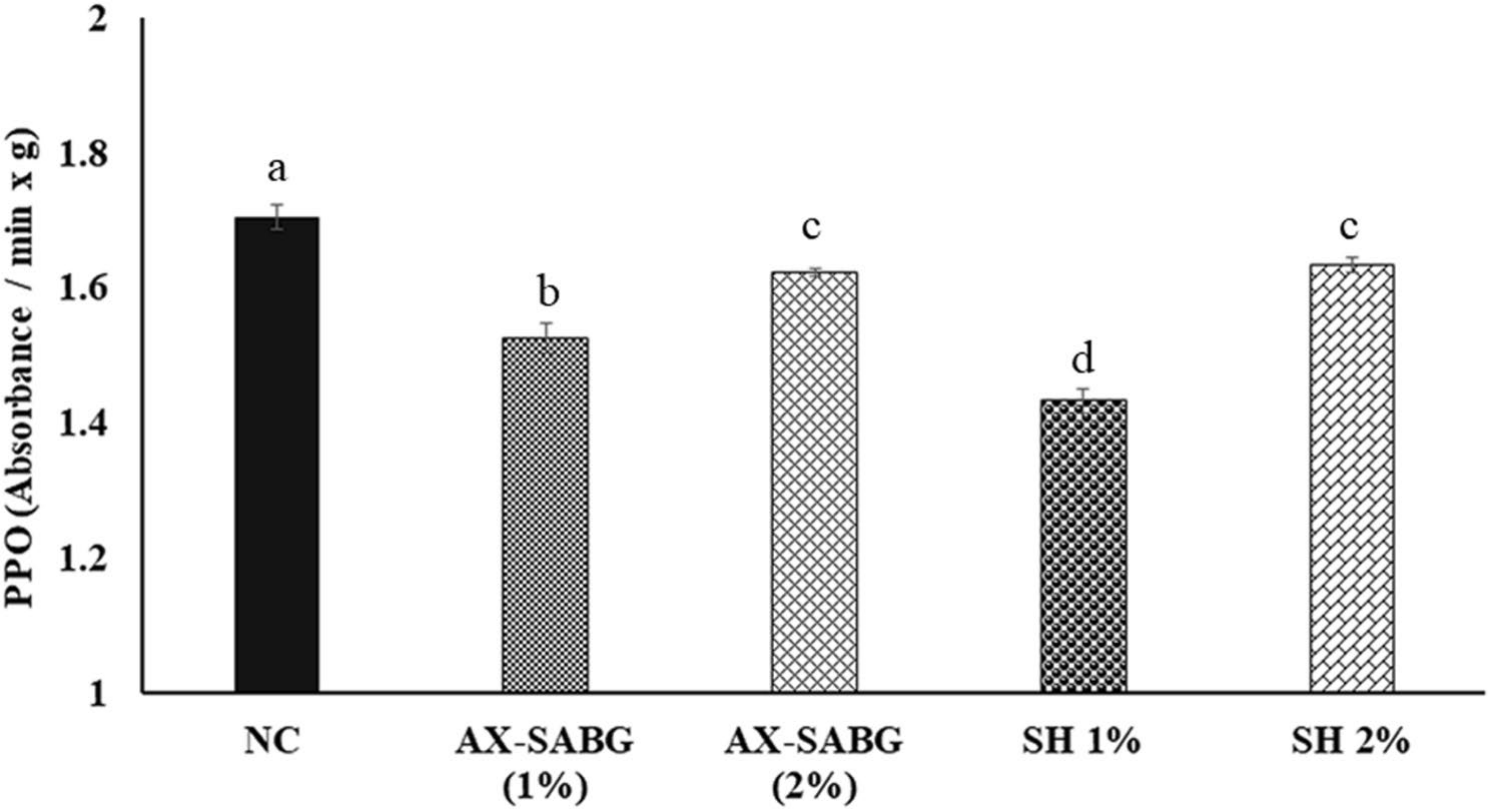
Change in polyphenol oxidase activity of peach during storage (*NC* uncoated, *AX-SABG* arabinoxylan-β-d-glucan stearic acid ester, *SH* shellac). Data were analysed using ANOVA followed by Tukey’s multiple comparisons tests. The letters compare changes between the treatments. Different letters indicate significant differences at *p* < 0.05.

The postharvest change in the color of fruit flesh are related to the oxidation of the phenolic compounds into quinines and their polymerization to brown pigments^50^. The PPO activity was increased in both coated and uncoated peaches (Fig. 4) as compared to fresh peach. The AX-SABG (1–2%) and shellac (1–2%) coated peaches showed significantly reduced (*P* < *0.05*) enzymatic activity as compared to uncoated peaches during 6 days storage. Indeed, at the end of the storage the AX-SABG and shellac (1–2%) coated peaches showed 4.8–10.5% and 4.2–15.9% reduction respectively in the enzymatic activity as compared to uncoated peach. The results are in agreements with the previous study that control peach showed significant increase in the PPO activity during storage^51^. These result demonstrated that the surface coating of peaches effective in reducing the enzymatic activity during storage.

### Volatile compounds

**Figure 5:**
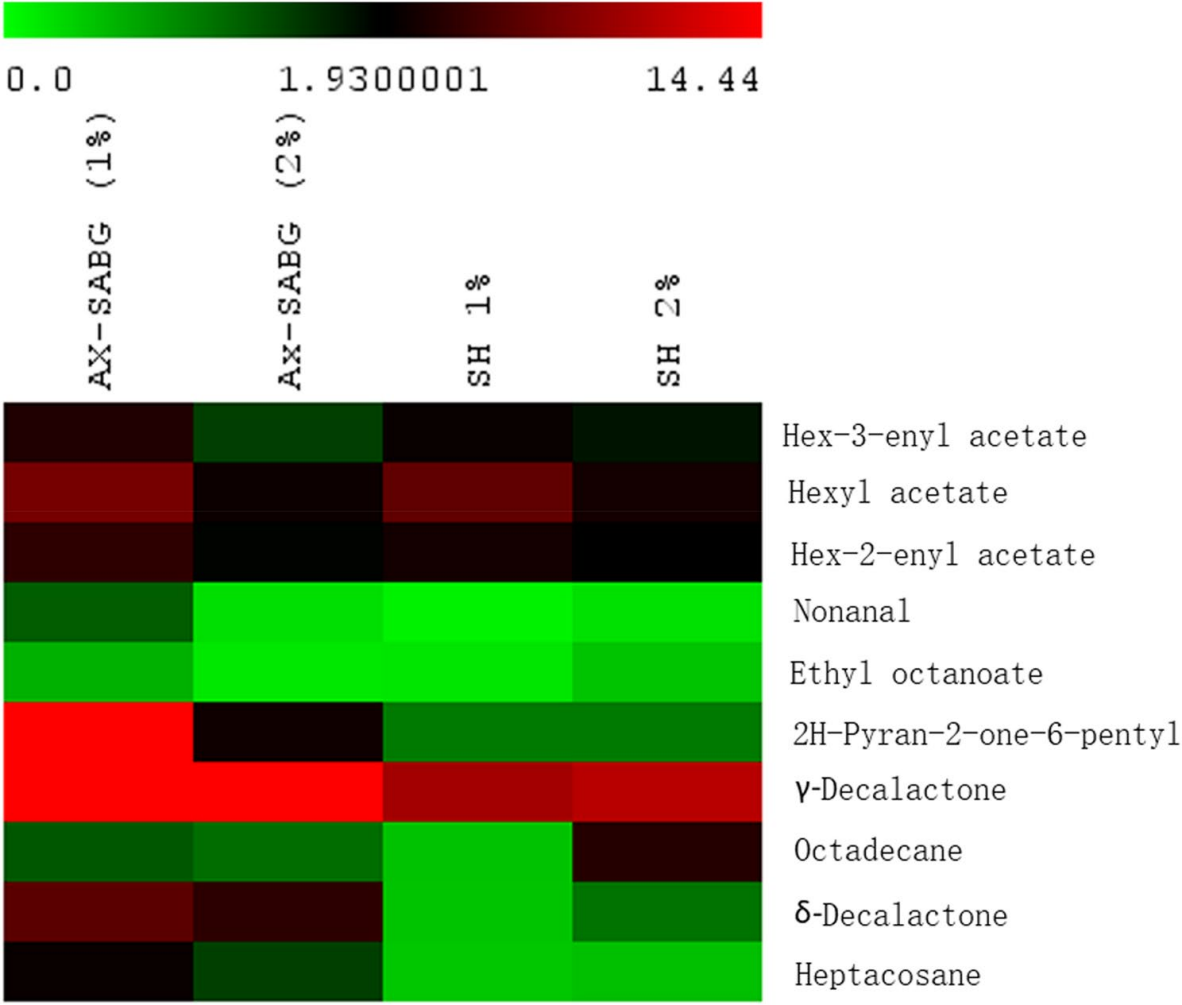
Distribution of volatile compounds in the AX-SABG and shellac (1–2%) coated peaches during storage (*AX-SABG* arabinoxylan-β-d-glucan stearic acid ester, *SH* shellac).

During 6 days storage lactones (δ-Decalactone, γ-Decalactone and 2H-Pyran-2-one-6-pentyl) and hexyl esters (Hex-3-enyl acetate, Hexyl acetate, Hex-2-enyl acetate) were identified as major aroma volatile compounds. Minor aroma volatile compounds such as Ethyl octanoate, Octadecane, Nonanal, and Heptacosane were also detected under similar storage. The effect of AX-SABG and shellac coating on volatile compounds of peaches during storage are presented in Fig. 5 and Supplementary Fig. 1. The concentrations of total volatile compounds were found significantly higher (45.6–99.5 µg/kg) in AX-SABG (1–2%) coated peaches compared to shellac (1–2%) coated fruits (24.4–26.4 µg/kg).

It has been previously reported that lactones particularly γ and δ-Decalactones were the major contributors to impact characteristic volatile compounds for peach and nectarine aroma^52,53^. During storage concentration of γ and δ-Decalactones were remarkably higher (27.1–47.4 µg/kg and 4.2–6.4 µg/kg respectively) in AX-SABG (1–2%) coated peaches. Whereas, lower concentrations (10-11 µg/kg and 0.4–1.1 µg/kg respectively) were measured in shellac coated fruits. Similarly, AX-SABG (1–2%) coated peaches had notably higher concentration of 2H-Pyran-2-one-6-pentyl in the range of 2.8–21.7 µg/kg whereas it was nearly undetectable in shellac coated fruits. Slightly higher concentrations of hexyl esters were also detected in AX-SABG coated peaches compared to shellac coated fruits. The overall results suggested that AX-SABG coating were effective in reducing metabolic activity and retaining volatile during 6 days storage. Further several studies have reported that the high membrane fluidity might be responsible for the reduction of volatiles compounds in shellac coated peaches during storage^18,54,55^.

### Acute oral toxicity study

Acute oral toxicity studies in male mice showed no mortality or treatment related complications or any signs of toxicity. There was no significant change in mean body weight (bw), average feed intake, average water intake and liver weight in different groups of animals (Supplementary Fig. 2). At tissue level, there were no treatment related histological changes observed in any of the treatment groups even at the highest dose of 5000 mg/kg bw suggesting the non-toxic nature of AX-SABG (Supplementary Fig. 3). Thus, LD_50_ value of AX-SABG exceeds 5000 mg/kg bw and falls in GHS category 5. In accordance with Hodge and Sterner scale, the coating material is practically non-toxic.

### Sub-acute oral toxicity study

All the treated animals were in good health with no signs of morbidity or mortality or unusual behaviour throughout the study. Mean body weights in all the experimental groups was not significantly different (*P* > 0.05) as compared to the control group. The weight of control group indicating that daily ingestion of coating material had no effect on the mice body weight (Supplementary Fig. 4).

**Figure 6:**
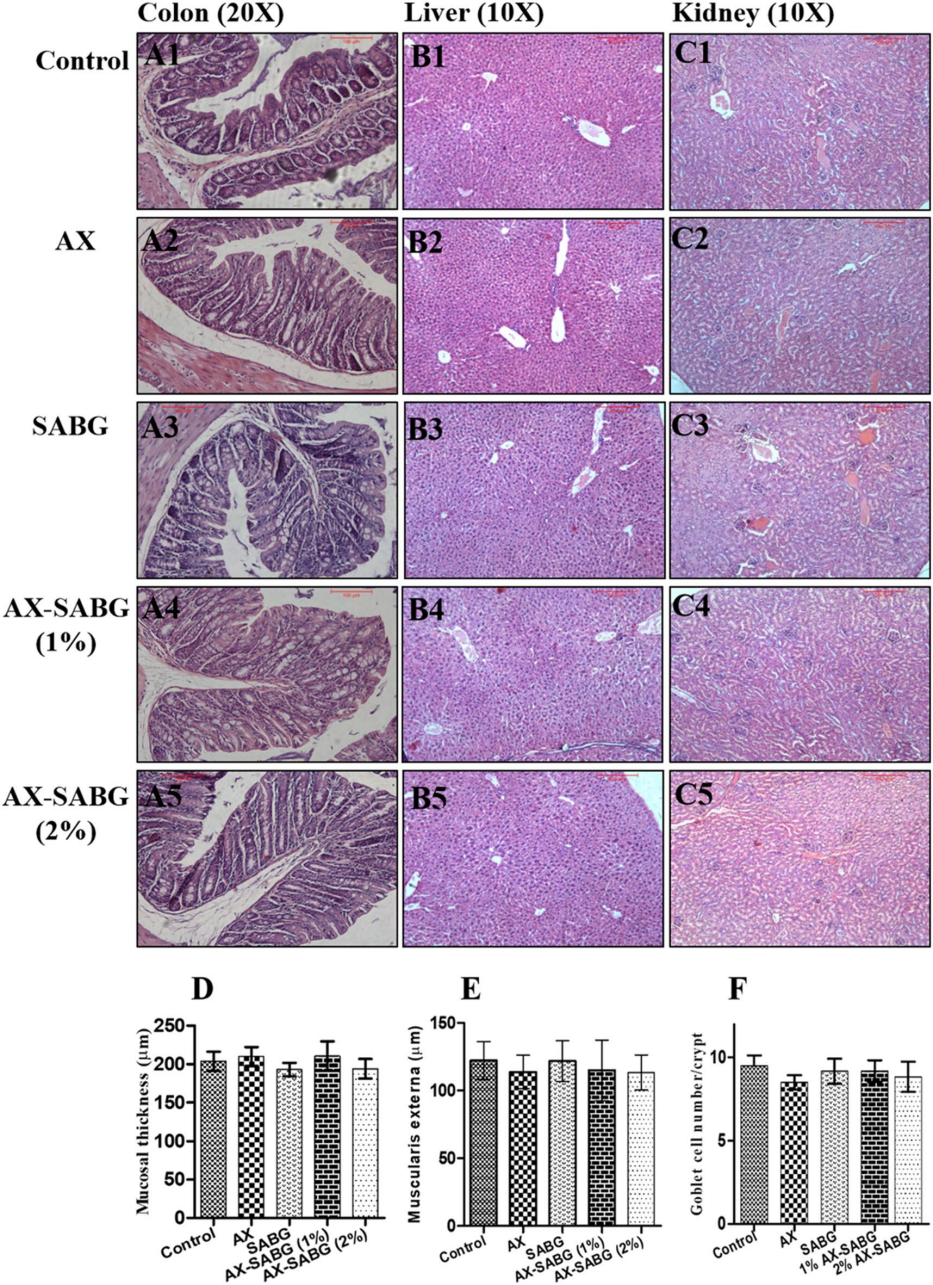
Representative images of histological sections of 28 day sub-acute oral toxicity study on colon **(A1–A5)** at ×20, liver **(B1–B5)** at ×10 and kidney **(C1–C5)** at ×10 of the control and treated groups and the graphs revealing **(D)** mucosal thickness, **(E)** muscularis thickness and **(F)** goblet cell count/crypt in the colon region of the control and treated groups (AX, SABG, 1% AX-SABG and 2% AX-SABG) (*AX* arabinoxylan, *SABG* β-d-glucan stearic acid ester).

Based on macroscopic observation, no obvious change in the appearance or size of the colon, liver and kidney were observed in any of the groups. None of the mice showed any signs of colon enlargement or hepatomegaly. Even at the microscopy level, no architectural abnormalities were found in the different tissues revealing the non-toxic nature of the coating material (Fig. 6). Macrophage infiltration was not observed in any of the animal tissues. The specific colonic features which undergo alterations in case of the inflammation such as reduction in muscularis externa thickness, mucosal length or goblet cell count were absent in all treated groups. There was statistically insignificant change (*P* > 0.05) in these parameters among the different groups of the mice (Fig. 6D–F). Parallel results were evident in the liver and kidney tissue sections. The morphology of both the tissues was intact with no signs of neutrophil infiltration (Fig. 6B,C). The hepatocytes and portal tracts in liver and renal glomeruli and tubules in kidney were found to be normal in all the experimental groups. Overall, the histological investigation confirmed that the coating material is non-toxic, non-inflammatory and safe for human consumption.

## Conclusion

The present study examined the effect of AX-SABG (1–2%) and shellac (1–2%) coating on postharvest quality of peach (*Sharbati*). The study revealed both AX-SABG and shellac (1–2%) had nearly similar efficacy in reducing weight loss, ripening index and maintaining fruit firmness. However, AX-SABG coating showed more pronounced effect compared to shellac in retaining color characteristics and aroma volatiles in fruits. The overall result suggested that AX-SABG (1–2%) was effective in maintaining fruit quality during 6 days storage under supermarket condition. The present study further suggested that AX-SABG coating has the potential to be alternative to animal derived shellac coating in India. The coating improves the quality and post-harvest storage life of peaches specifically during transportation and storage. Further, no tissue related toxicity and mortality was observed in both acute and chronic toxicological studies suggesting AX-SABG is non-toxic and safe for human consumption.

## Supplementary Information

Supplementary Figures.
