# Supplementary material for: Application and toxicity studies of arabinoxylan and β-d-glucan stearic acid ester composite coatings in extending postharvest storage of peach
Source: Sci Rep. 2021 Dec 13;11:23848. doi: 10.1038/s41598-021-03163-5 (PMC8668965; doi:10.1038/s41598-021-03163-5)
Supplement: Supplementary file 1 — Supplementary Figures. [file 41598_2021_3163_MOESM1_ESM.docx]

**Application and toxicity studies of arabinoxylan and β-D-glucan stearic acid ester composite coatings in extending postharvest storage of peach**

Usman Ali^a^, Prabhjot Kaur^a #^, Swati Kanwar^a^, Vibhu Kumar^a^, Rohit Maurya^a^, Mahendra Bishnoi^a^, Santanu Basu^b^, Koushik Mazumder ^a^ *

^a^National Agri-Food Biotechnology Institute, Sector-81 (Knowledge City), S.A.S. Nagar, Mohali-140306, Punjab, India.

**^b^**Department of Molecular Sciences, Swedish University of Agricultural Sciences, P.O. Box 7015, SE-750 07, Sweden

^#^Contributed equally

*Corresponding author: Tel.: +91-172-5221244, Fax: +91-172-5221100, E-mail address: koushik@nabi.res.in, [kmazumder78@gmail.com](mailto:kmazumder78@gmail.com)

**Supplementary Figures**

**
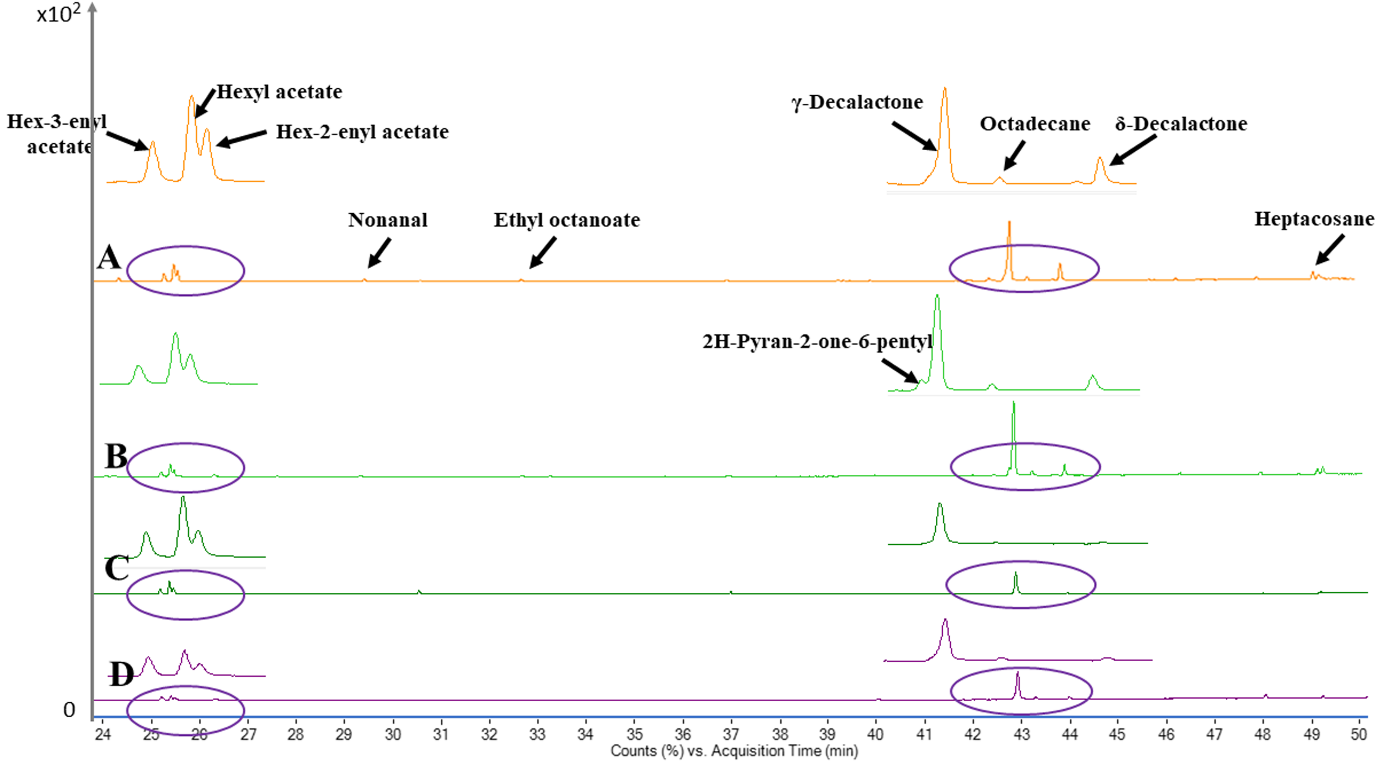
**

**Supplementary Fig. 1.** GC-MS chromatogram of peach volatiles compounds during storage (A- AX-SABG 1%, B- AX-SABG 2%, C- SH 1% and D- SH 2%)

**B**

**C**

**D**

**A**

**Supplementary Fig. 2.** Acute Toxicology Study: A) Mean Body weight (bw) changes, B) Liver weight, C) Water intake and D) Feed intake per cage over 24 hour’s interval per cage (n=6) in control and AX-SABG treated (50-5000mg/kg bodyweight) groups.


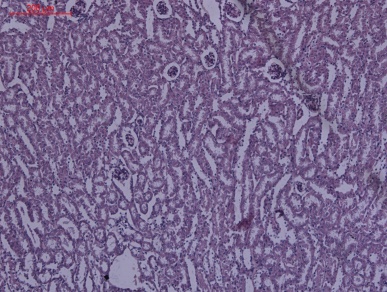

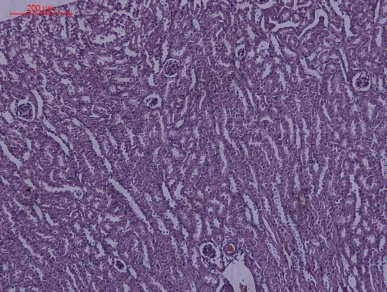

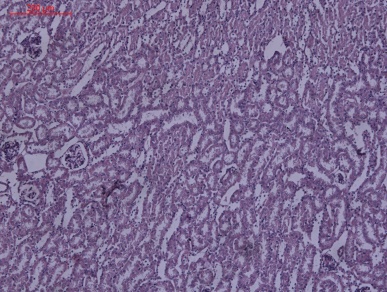

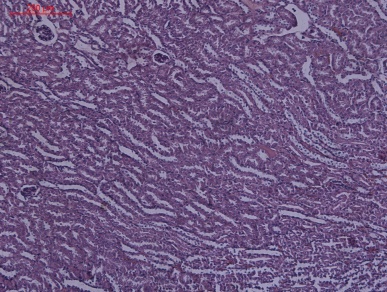

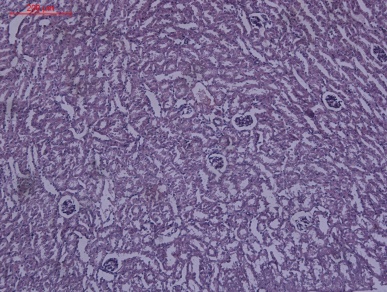

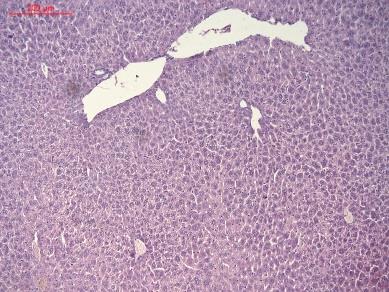

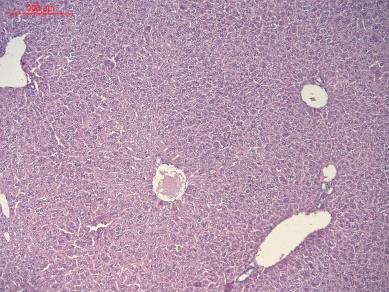

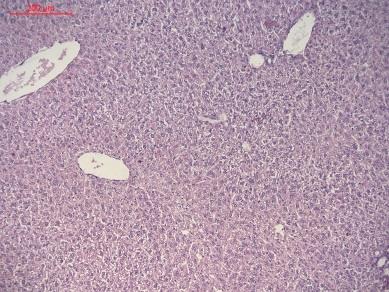

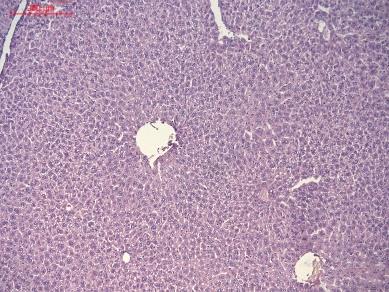

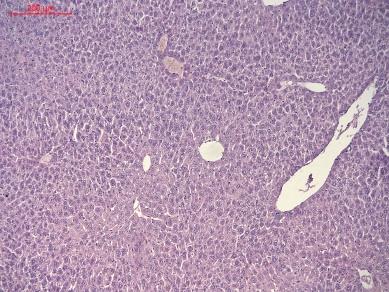

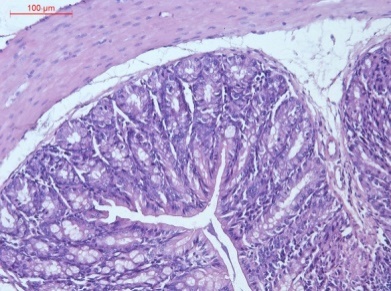

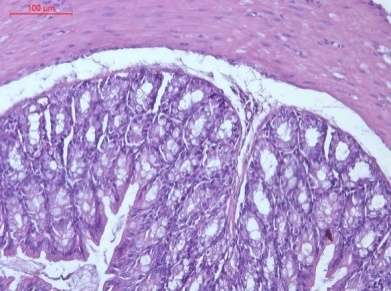

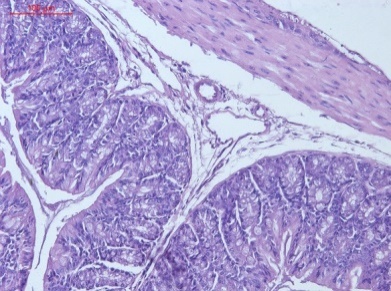

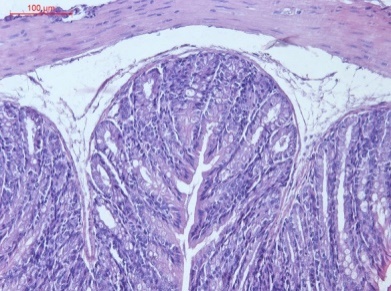

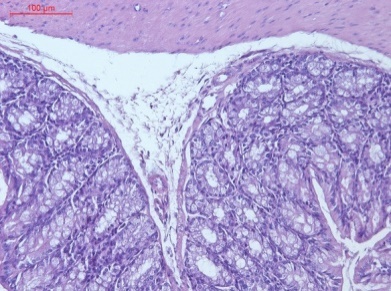


**D**

**E**

**F**

Kidney (10X)

Liver (10X)

Control

AX-SABG

50mg/kg bw

AX-SABG

2000mg/kg bw

AX-SABG

300mg/kg bw

AX-SABG

5000mg/kg bw

Colon (20X)

A1

A3

A2

A5

A4

B1

B3

B2

B5

B4

C1

C5

C2

C3

C4

**Supplementary Fig. 3.** Representative images of histological sections of 14 day acute oral toxicity study on colon (A1-A5) at 20X, liver (B1-B5) at 10X and kidney (C1-C5) at 10X of the control and treated groups and the graphs revealing the Muscularis thickness (D), Mucosal thickness (E), Goblet cell count/crypt in the colon region of the control and treated groups (AX, SABG, 1% AX-SABG and 2% AX-SABG).

**Supplementary Fig. 4.** Sub-acute oral toxicity study: Mean body weights of the control and experimental groups (n=6) which were exposed to AX, SABG, 1% AX-SABG and 2% AX-SABG.
